# Supplementary material for: Epithelial cell chirality emerges through the dynamic concentric pattern of actomyosin cytoskeleton
Source: eLife. 2025 Jul 8;14:e102296. doi: 10.7554/eLife.102296 (PMC12387757; doi:10.7554/eLife.102296)
Supplement: Figure 2—figure supplement 2—source data 1. [file elife-102296-fig2-figsupp2-data1.pdf]

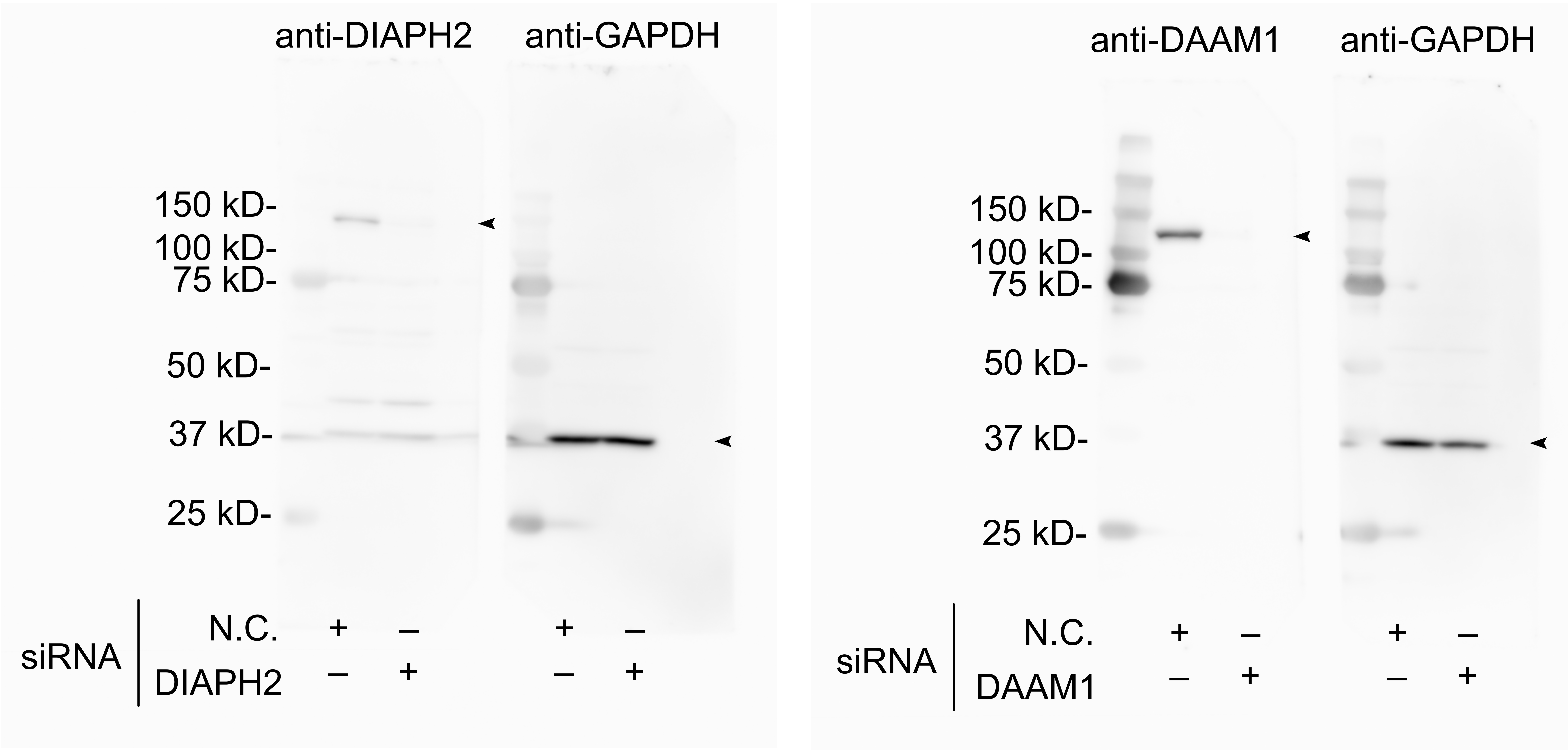

Figure 2 – figure supplement 2, Source Data 1. Original membranes corresponding to Figure2 – figure supplement 2B and C. siRNA treatments are indicated below each lane. Arrowheads mark the bands of interest. these bands are shown with contrast adjustment in Figure 2—figure supplement 2B–C.
