# Supplementary figures and images for: Epithelial cell chirality emerges through the dynamic concentric pattern of actomyosin cytoskeleton
Source: eLife. 2025 Jul 8;14:e102296. doi: 10.7554/eLife.102296 (PMC12387757; doi:10.7554/eLife.102296)

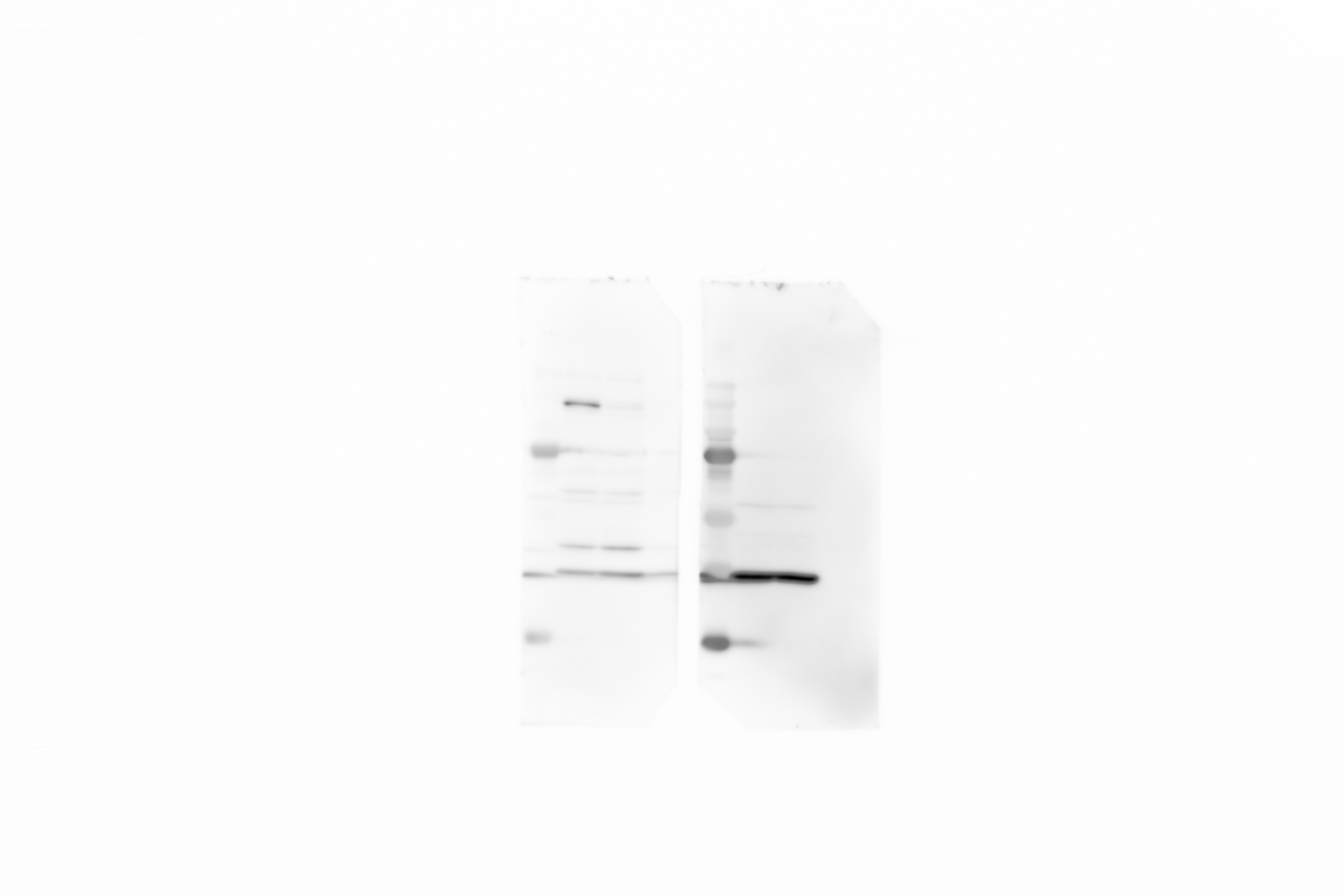

Supplement: Figure 2—figure supplement 2—source data 2. [file elife-102296-fig2-figsupp2-data2.zip › Figure 2-figure supplement 2-source data 2/OriginalRawUncroppedBlot_DIAPH2_GAPDH.tif]

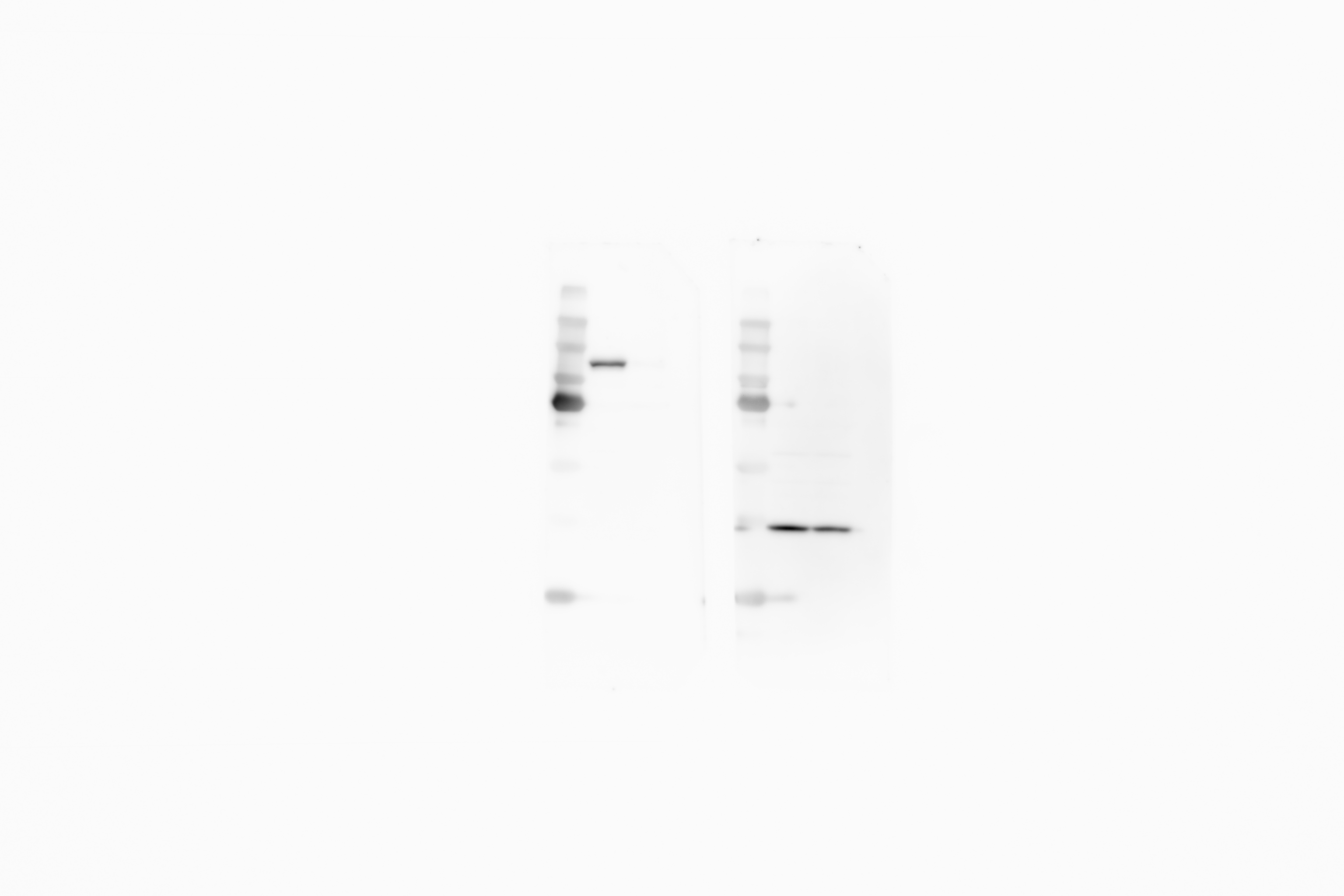

Supplement: Figure 2—figure supplement 2—source data 2. [file elife-102296-fig2-figsupp2-data2.zip › Figure 2-figure supplement 2-source data 2/OriginalRawUncroppedBlot_DAAM1_GAPDH.tif]

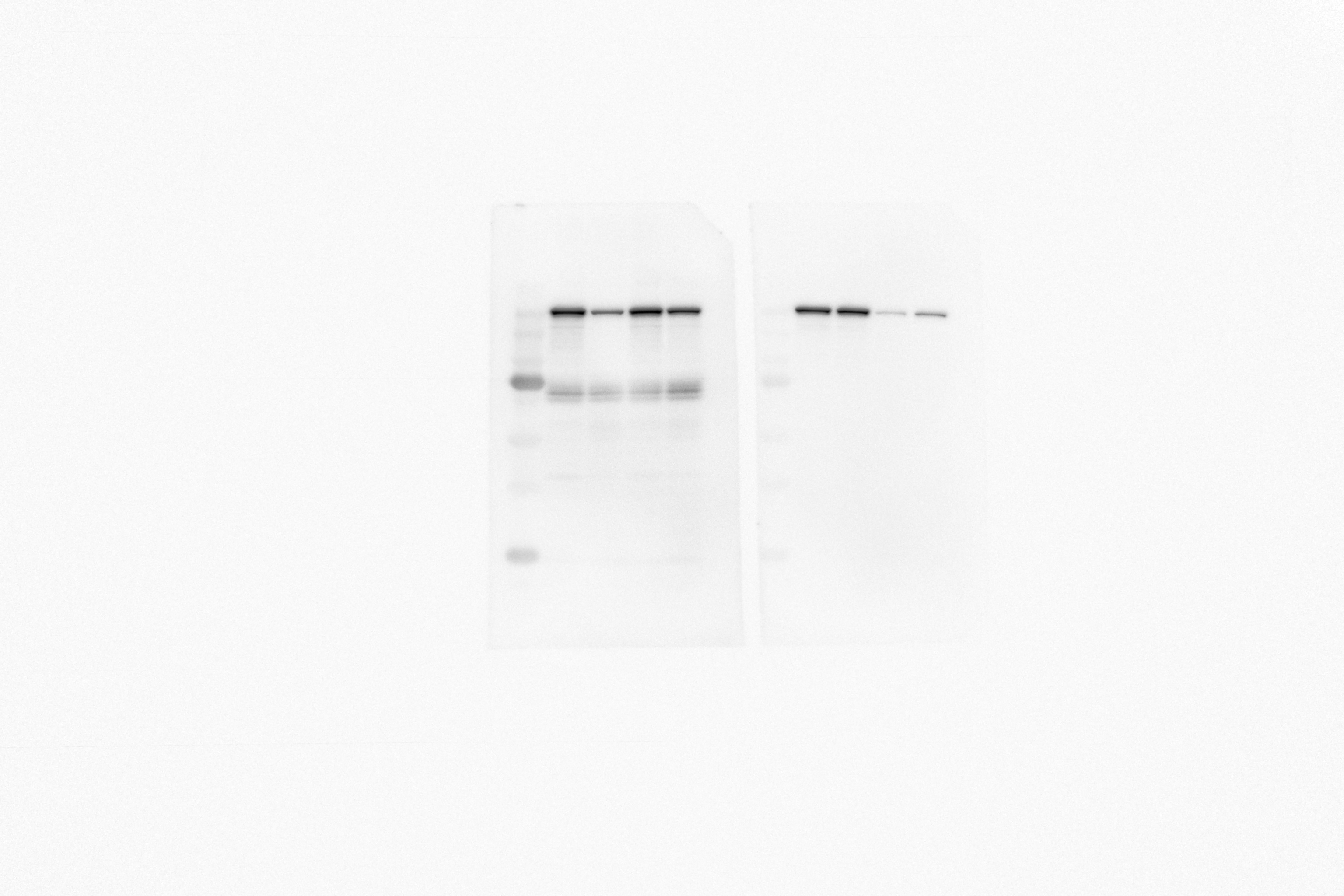

Supplement: Figure 2—figure supplement 3—source data 2. [file elife-102296-fig2-figsupp3-data2.zip › Figure 2-figure supplement 3-source data 2/OriginalRawUncroppedBlot_MyoIIAB.tif]

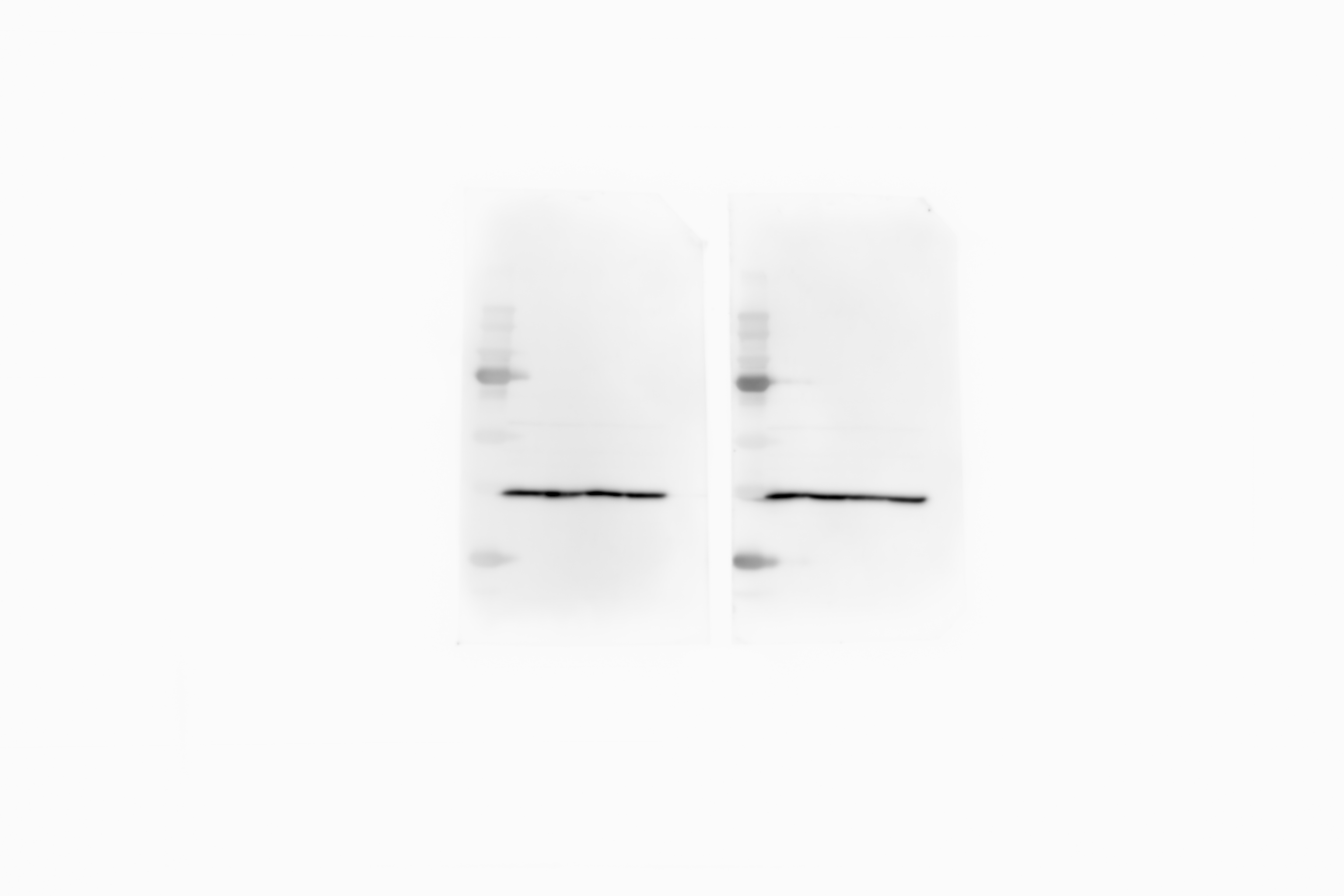

Supplement: Figure 2—figure supplement 3—source data 2. [file elife-102296-fig2-figsupp3-data2.zip › Figure 2-figure supplement 3-source data 2/OriginalRawUncroppedBlot_GAPDH.tif]

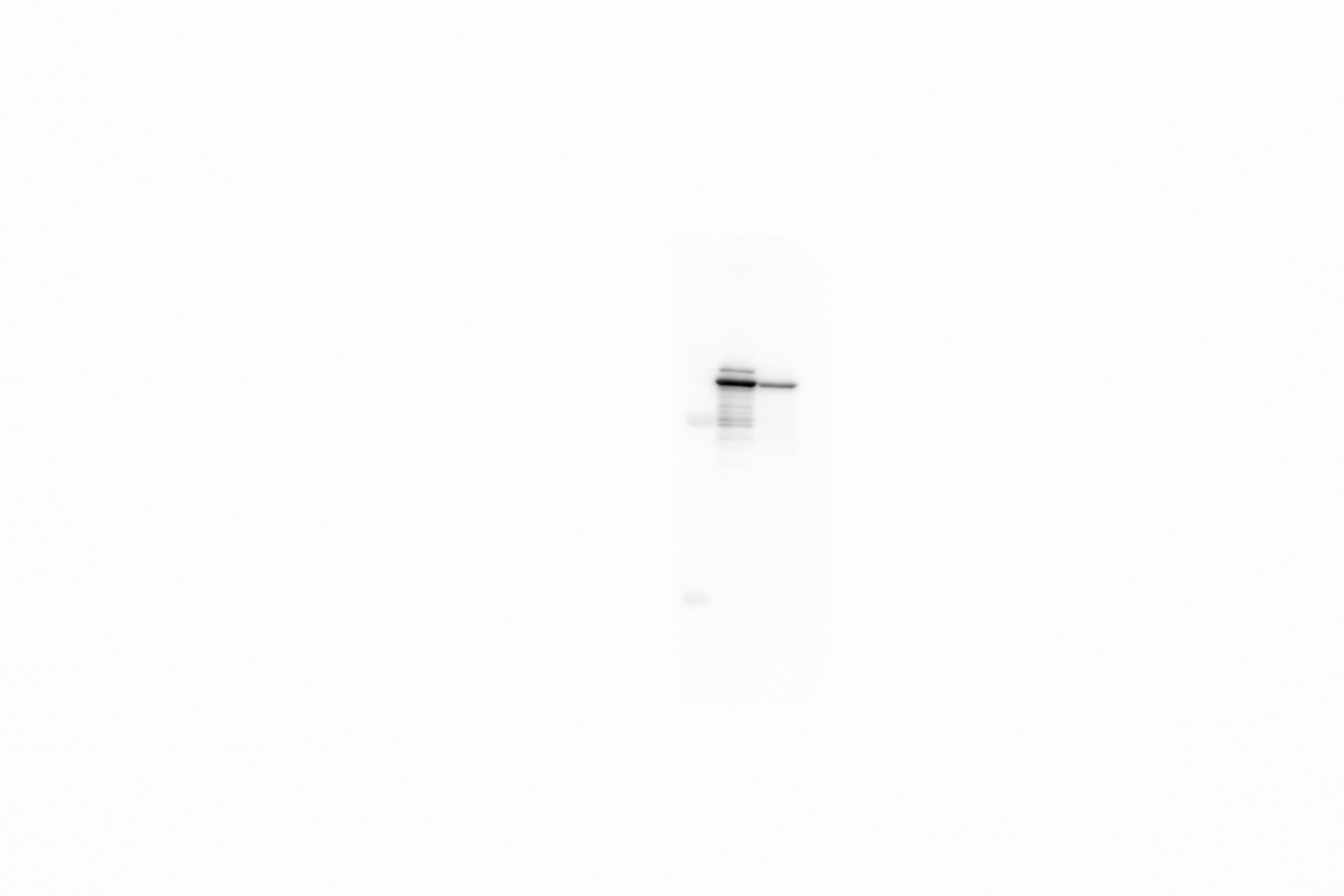

Supplement: Figure 2—figure supplement 4—source data 2. [file elife-102296-fig2-figsupp4-data2.zip › Figure 2-figure supplement 4-source data 2/OriginalRawUncroppedBlot_VCL.tif]

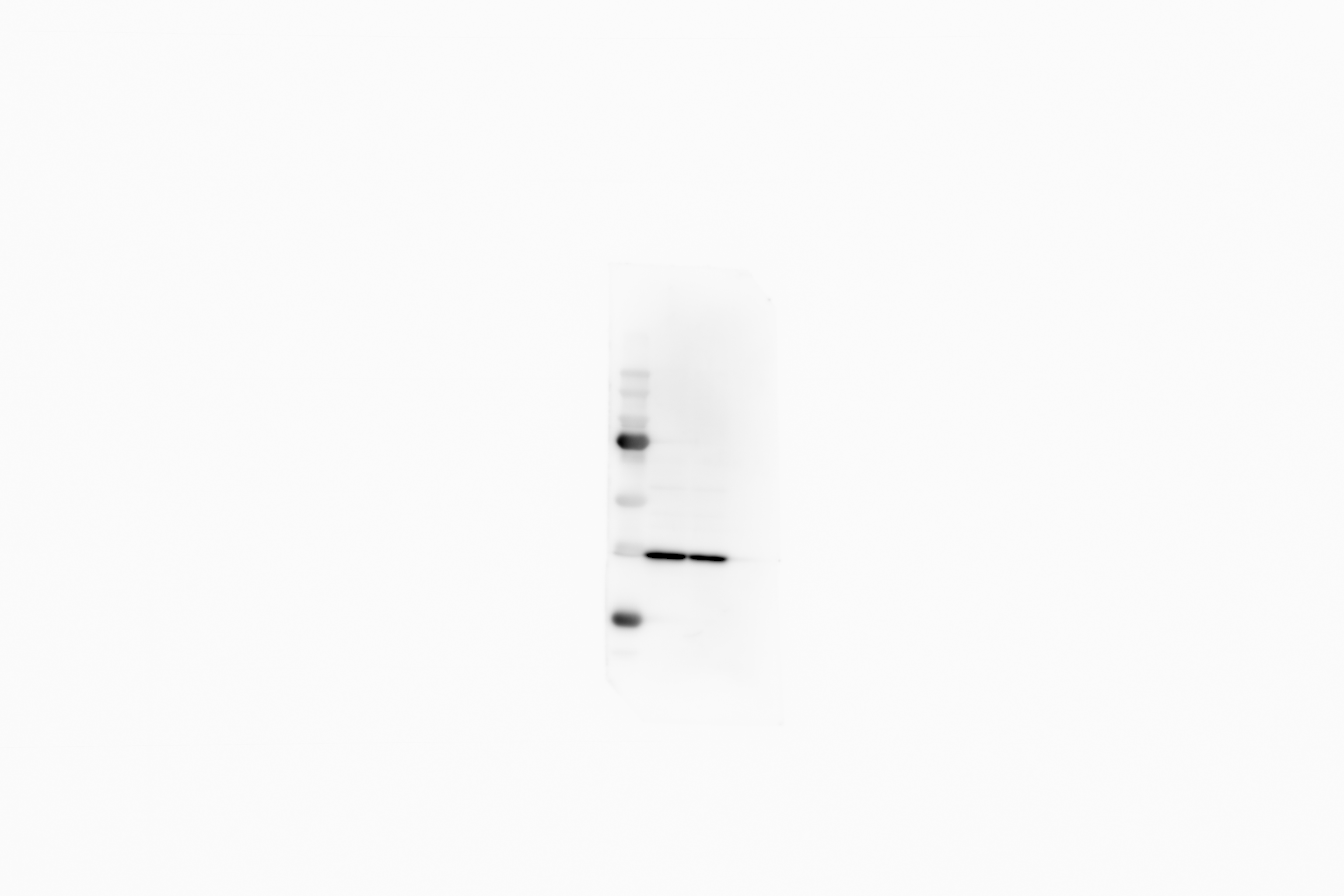

Supplement: Figure 2—figure supplement 4—source data 2. [file elife-102296-fig2-figsupp4-data2.zip › Figure 2-figure supplement 4-source data 2/OriginalRawUncroppedBlot_GAPDH.tif]
